# Redesigning the Paradigms of Clinical Practice for Oral and Maxillofacial Surgery in the Era of Lockdown for COVID-19: From Tradition to Telesemeiology

**DOI:** 10.3390/ijerph17186622

**Published:** 2020-09-11

**Authors:** Massimo Robiony, Elena Bocin, Salvatore Sembronio, Fabio Costa, Vittorio Bresadola, Alessandro Tel

**Affiliations:** 1 Department of Medicine, University of Udine, 33100 Udine, Italy; elena.bocin@gmail.com (E.B.); info@sembroniomaxillo.com (S.S.); drfabiocosta@libero.it (F.C.); vittorio.bresadola@uniud.it (V.B.); alessandro.tel@icloud.com (A.T.); 2 Maxillofacial Surgery Department, Academic Hospital of Udine, 33100 Udine, Italy; 3 Rehabilitation Unit, Academic Hospital of Udine, 33100 Udine, Italy; 4 Department and Simulation Center, Academic Hospital of Udine, 33100 Udine, Italy

**Keywords:** COVID-19, telemedicine, teleconsultation, telesemeiology, facial care project

## Abstract

The rise of the COVID-19 pandemic has posed new challenges for health care institutions. Restrictions imposed by local governments worldwide have compromised the mobility of patients and decreased the number of physicians in hospitals. Additional requirements in terms of medical staff security further limited the physical contact of doctors with their patients, thereby questioning the traditional methods of clinical examination. Our institution has developed an organization model to translate the essential clinical services into virtual consultation rooms using a telemedicine interface which is commonly available to patients. We provide examples of clinical activity for a maxillofacial surgery department based on teleconsultation. Our experience is summarized and an organization model is drafted in which outpatient consultation offices are translated into virtual room environments. Clinical examples are provided, demonstrating how each subspecialty of oral and maxillofacial surgery can benefit from virtual examinations. The concept of “telesemeiology” is introduced and a checklist is presented to guide clinicians to perform teleconsultations. This paper is intended to provide an organization model based on telemedicine for maxillofacial surgeons and aims to represent an aid for colleagues who are facing the pandemic in areas where lockdown limits the possibility of a physical examination.

## 1. Introduction

During December 2019, cases of pneumonia caused by an unknown viral agent were reported for the first time in the city of Wuhan, China. Subsequently, health care institutions have named that agent as “2019 novel coronavirus (2019-nCOV)” or COVID-19 [1]. In the following months, the world has seen a rapid spread of the disease, which has now infected almost 16 million people worldwide and caused approximately 640,000 deaths [2]. Italy has been seriously hit by the COVID-19 pandemic, with the first cases dating back to the end of February 2020. As of the first half of August, almost 250,000 cases and 35,000 deaths have been confirmed [3].

The majority of countries in the world have experienced a “lockdown” phase, which means promoting restrictive measures with the intent of limiting movements or activities in a community while maintaining only essential services. The Italian government announced a national lockdown, also known as “Phase 1”, which started on March 10th and allowed only basic activities to take place. As the epidemiological trend raised optimistic predictions, on May 4th, the Italian government issued so-called “Phase 2”, which abolished many of the restrictions imposed by Phase 1 and allowed a higher circulation of people, for instance, permitting them to meet relatives and families and to practice sports activities. Such a transition intrinsically bore the risk of a new wave of infections, which were controlled by adopting the precautions and the protocols learnt in Phase 1. On May 18th, the Italian government planned a significant reduction of the imposed limitations, and this was referred to as “Phase 3”, which is the most delicate and worrying, unless it is accompanied by adequate precautions and correct behavioral rules. This has to necessarily start in the health care field, not just because health workers are the most exposed professional figures, but also because they have the knowledge and the ethical commitment to follow rules, apply protocols and keep updated.

During Phase 1, the overwhelming number of patients with severe respiratory symptoms requiring hospitalization and ventilation in ICU units put Italian health care systems under unprecedented stress, with all departments being forced to postpone the treatment of non-emergency patients during this crisis. Many countries in the world are currently facing the same situation, which from a clinical point of view means that no elective surgery should be performed, and even urgent procedures might be delayed until a nasopharyngeal swab is available. Logically, emergency procedures are beyond this logic, and even in the absence of a diagnostic test, the patient is considered positive until the contrary is demonstrated. The result is that during lockdown, clinical activity is essentially limited to life-saving procedures, to the detriment of routine services, especially daily outpatient consultations.

Besides, head and neck specialists, including oral and maxillofacial surgeons, dentists and ENT surgeons, are exposed to significant risk of virus transmission [4] and in countries under lockdown, this represents an additional reason for which many patients may suffer from a restricted access to outpatient clinical evaluation. As a consequence, limitations posed by lockdown for patients needing an oral and maxillofacial surgery consultation might cause a diagnostic delay, both because there are inferior opportunities for patients to undergo clinical examination and because patients themselves are less motivated to search for clinical aid.

With the emergence of COVID-19, health care delivery has seen the rise of telemedicine services in the attempt to decrease the spread of COVID-19, protect medical and nursing staff and avoid depletion of personal protective equipment, such as gloves, masks and face shields [5].

Our oral and maxillofacial surgery department has further developed a previously presented telemedicine platform [6], which during the COVID-19 era has been reorganized into a versatile and scalable unit which integrates telemedicine services and provides remote assistance for clinical examinations. Collecting our experience learnt from Phase 1 lockdown [7], our aim is to provide a model to reorganize the routine services provided by a modern oral and maxillofacial surgery department using the support of telemedicine. This paper aims to be a guide and a help in a critical time to suggest a strategy to convert traditional health care into virtual teleassistance.

## 2. Materials and Methods

### 2.1. Technological Core for Videoconsultation

The basic hardware requirements for a simple teleconsultation setup consisted of a computer connected to a digital camera, commonly referred to as a “webcam”, speakers and a microphone. All the latest generation laptops embed all the required tools. Similarly, in case a smartphone or a tablet is used, portable devices also integrate a camera. Before being scheduled for the videoconference examination, the patient was administered a questionnaire assessing the availability of technological devices and, especially for elderly people, the presence of a more skilled person to provide help during the setup phases, as well as previous experience with teleconference systems.

The communication protocol for the telematic examination of patients was based on Zoom software (Zoom Video Communications Inc, San Jose, CA, USA). Both the clinician and the patient were required to install a copy of the software, which can be downloaded free of charge. The software is available for multiple operating systems, including Windows and Mac, as well as for mobile devices, since both iOS and Android are supported. This software resulted in a very versatile solution because in its free version it allows hosting of up to 100 participants for a meeting of a maximum of 40 min. For clinical use, our institution has purchased a license which indefinitely extends the meeting time, even though 40 min are sufficient for almost all teleconsultations. Zoom presents a complete toolset with many useful features: remarkably, it allows screen sharing, including a portion of it, or even single applications; additionally, it enables remote control of the screen, a useful feature to assist patients with fewer technological skills, or to indicate specific details, for instance, when analyzing complex radiological data. It is also possible to assign every Zoom account a unique ID, which is a univocal code composed of 10 characters. In this way, the user creates his own meeting space, a virtual room environment (VRE), which can be used for videoconferences. In our experience, this resulted in the creation of the room “Maxillofacial Surgery Clinic Examination Office”, which was the way it appeared on the patient’s screen. This study did not require IRB approval since it did not modify in any way patients’ treatment and it did not introduce novel technologies.

### 2.2. Reorganization of Clinical Activity

We created a protocol which was distributed to patients scheduled for an outpatient maxillofacial consultation as a PDF file, where the patient could find the download links, as well as the room code. The patient awaited his or her time to undergo the virtual examination and, a few minutes before, a phone call was made communicating the password for the meeting. In the meantime, the patient had the possibility to properly configure the software, microphone and webcam. Once the patient entered the communicated password, he or she was admitted into the waiting room, which was a useful feature in case the previous examination was still going on. As the previous patient concluded his examination, the physician accepted the new patient’s request and provided admission to the virtual space. Zoom allowed us to record the full video session, which was stored for each examination as an official document. The clinical database was compiled as if a physical examination were performed and another date was communicated to the patient if a follow-up was needed.

### 2.3. Activities Made Possible by the Virtual Room Environment (VRE)

According to our protocol, the VRE allowed for multiple activities to take place with the aid of telemedicine. Each activity is briefly described as follows:

#### 2.3.1. Morning Meeting and Multidisciplinary Meeting to Organize Complex Surgery

**Figure 1:**
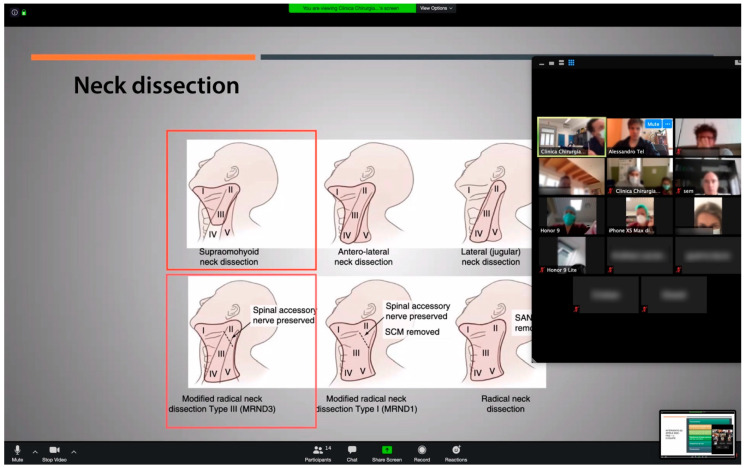
Multidisciplinary meeting with multiple participants, including anesthesiology team. A case of oncological surgery is discussed using Zoom.

A Virtual meeting allowed the medical team to meet every morning to discuss clinical cases and to take stock of the week’s organization. For complex oncologic surgeries, a multidisciplinary meeting could be organized with the remote participation of other surgical specialists, anesthesiologists, nurses and rehabilitation staff (Figure 1).

Each clinician presented his or her clinical case by sharing his or her screen and displaying related images. The head of department hosted the meeting session and residents wrote down a final record which was shared with all the participants. Additionally, the nursing staff and the administrative personnel with the task of organizing appointments for patients were part of the meeting.

#### 2.3.2. Examination of Patients

**Figure 2:**
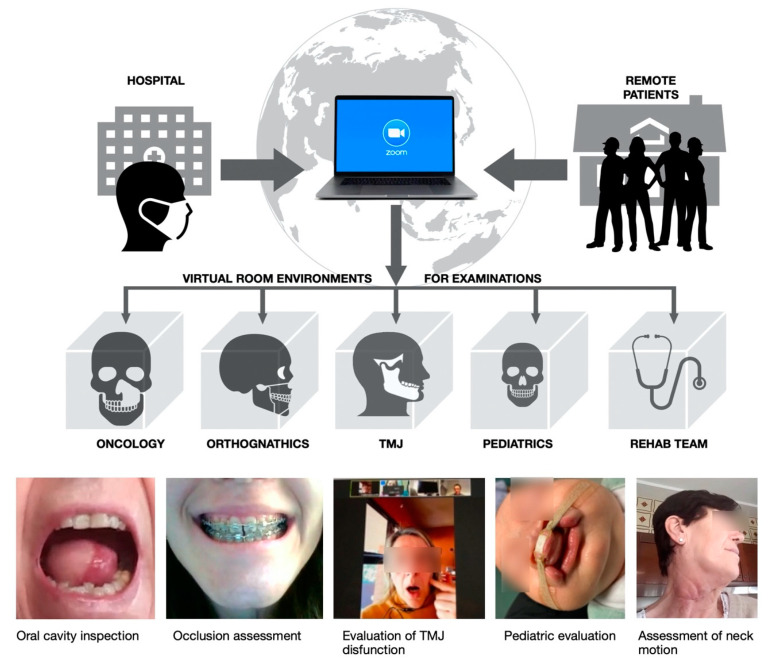
Organization of consultation rooms into VREs and clinical examples.

In our department organization, outpatient consultations include a first-level general maxillofacial evaluation, after which patients have access to second-level specialist consultations. The access to specialist examination rooms is direct for post-operative patients. Such organization was maintained unaltered and virtually translated into a digital environment, in which VREs replaced physical rooms. The patient was given access with the univocal Zoom ID which identified the consultation rooms. As a consequence, the following consultation VREs were designed: orthognathics; temporomandibular joints; pediatric maxillofacial surgery, oncology. Additionally, a particular VRE for rehabilitation medicine was created in which a rehabilitation physician and physiotherapists performed a postoperative assessment for recently dismissed patients who previously underwent complex surgeries consisting in demolitive surgery and reconstruction with free flaps. The organization of virtual examination rooms is schematically shown by Figure 2.

Moreover, the hospital network at our institution was empowered by clinical engineers to provide the possibility to remotely access clinical records and databases, allowing us to instantly check the situation of each hospitalized patient including when the clinician was not in the hospital.

#### 2.3.3. Surgery

**Figure 3:**
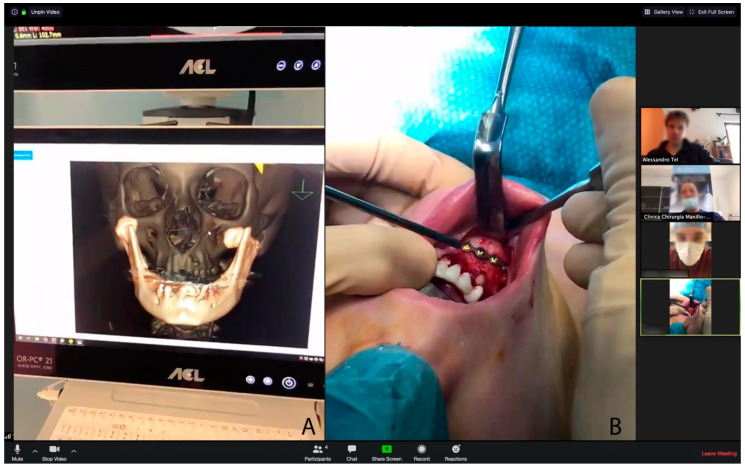
Videosurgery transmitted from the operating theatre. (**A**) Radiology images are viewed using an online consultation system. The presented image portrays a case of left condylar fracture associated to a right parasymphiseal fracture. (**B**) Surgical phases are conducted under the virtual guidance of a more expert surgeon. This phase shows the synthesis of the parasymphyseal fracture.

The use of telementoring was proven to be effective during surgical sessions performed in the lockdown. The COVID-19 pandemic outbreak restricted us to performing only trauma and oncologic surgeries, while elective procedures were postponed indefinitely. A laptop was installed in the operating room and the Zoom application was properly configured. Using only low-cost and mobile technology [6], a network between the operating room with surgeons on shift and remote surgeons was set up (Figure 3).

This organization allowed us to maintain the support of the entire medical staff, since, in case of need, an experienced surgeon could remotely provide assistance to the younger surgeon in the operating room, as described by Bhattarai et al. [8].

Moreover, the COVID-19 pandemic gave new impetus to scientific research, as restrictions on medical wards provided more time to dedicate to this important activity. Teleconference systems allowed us to perform scientific meeting sessions, in which every participant shared his or her own ideas about the research trends and discussed proposals for scientific papers. Similarly, the activity of paper correction and data analysis became more prominent in researchers employing their time to write papers.

#### 2.3.4. Medical Aid in COVID-19 Areas

A particular form of assistance clinician was called to provide support in critically saturated departments for COVID-19 patients, like the infectious diseases department. In this setting, the role of the oral and maxillofacial surgeon was to offer a general medical knowledge to provide information to patients via telemedicine in order to filter access and to establish which patients needed hospitalization according to their clinical features. As our team has been using telemedicine for a long time, the presence of oral and maxillofacial surgeons in such departments allowed us to introduce their experience in the use of telemedicine, providing the whole medical and nursing staff with the know-how to connect via Zoom or Skype with patients and their families using tablets.

## 3. Results

In the presence of the appropriate technological devices and a suitable internet connection, the examinations were successfully conducted for all patients. A checklist was prepared in order to synthetically collect all the necessary information from recently discharged patients who previously underwent surgery. This “telesemeiology checklist” covered the many different types of procedures by creating common questions exploring the different aspects of pain, swelling, fever and neurosensory disturbances. More specific questions were conceived for orthognathic surgery, TMJ surgery, oncology and trauma. Subsequently, inspective examination was conducted, by repeating some of the same steps, such as occlusion evaluation, mouth opening and facial movements to assess the functionality of the facial nerve. The presented checklist is shown in Table 1.

During the lockdown, four diagnoses of malignancies were suspected in the VRE dedicated to oral and maxillofacial oncology. Patients underwent subsequent diagnostic investigation and were invited for a traditional examination and then for a biopsy: three patients had moderate to severe dysplasia, while one diagnosis of oral squamocellular carcinoma was confirmed.

At the end of the procedure, each patient was administered a satisfaction survey, whose results were collected and delivered to the hospital director (Table 2).

During Phase 1 lockdown, from March 11th to April 20th, 78 patients at our institution were clinically evaluated using teleconsultation. Fifty patients had been operated on before the COVID-19 era and needed postoperative evaluation, but were unable to move because of restrictions, while 28 patients needed non-deferrable examinations, and were remotely assisted. All patients were administered the satisfaction questionnaire after teleconsultation, whose results were collected. Eighty-two percent of patients reported an optimal experience in using teleconsultations (score from 13 to 17), 13% of patients reported a good experience (from 8 to 13), while according to 5% of patients, the experience was unsatisfactory.

## 4. Discussion

In this rapidly evolving crisis, flexibility plays a key role in health care institutions and is required from both doctors and patients. Similarly, this pandemic offers the opportunity to study and develop innovative solutions to overcome the problem of restrictions imposed by the lockdown. The challenge for a modern health care institution is to maintain a high quality of care, at the same time safeguarding both patients and clinicians by reducing the number of physical consultations. During this time, we have seen a chaotic flow of patients overwhelming the hospital departments. For such reasons, our hospitals have canceled or deferred all non-essential outpatient consultations to reduce the risk of cross-infection. Telemedicine allowed us to bridge the gap in clinical care caused by the decrease in outpatient consultations, allowing us to intercept suspected pathologies.

Many other specialties have experienced similar issues in the COVID-19 era, such as otolaryngology [9], orthopedics [10], urology [11] and diabetology [12]. Many protocols have also been developed for oral and maxillofacial surgery, as described by Zimmermann et al. [13], Ebben et al. [14], Blomestrand et al. [15] and Brockes et al. [16]. Telemedicine was also used to conduct clinical examinations on pediatric patients, as described by the work of Farook et al. [17]. Additionally, telemedicine played a crucial role in sharing radiological images, a useful feature when discussing a clinical case or performing a surgical procedure, for instance, a case of maxillofacial trauma [18], as shown in Figure 3.

Telemedicine has provided a pathway to overcome all the mentioned obstacles, and has allowed for a fluent management of emergencies, minimizing the risk of exposure of health care providers as well as decreasing their workload, given the continuous request for medical personnel in departments such as infectious diseases, pneumonology and anesthesiology. At the same time, telemedicine services continue to play a key role to maintain the continuity of care and to bring assistance to remote areas [19].

Moreover, the foreseeable future gives a glimpse of the importance of collecting clinical data to improve patients’ care, especially considering the growing field of big data analysis. In this respect, videoconsultations offer a great opportunity to refine our ability to capture clinical signs of disease in remote patients. As a consequence, telemedicine opened the doors to a novel “telesemeiology” concept, which may play an increasingly important role in the future to perform distant consultations in remote areas or to perform a basic screening of patients on the basis of their clinical findings in order to decrease waiting lists for a first examination.

**Figure 4:**
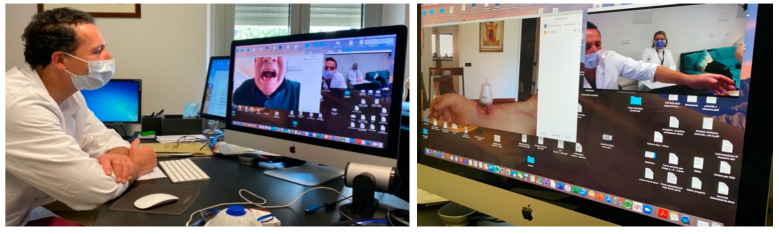
Dedicated sessions are performed for complex oncological patients. The left is Inspection of the oral cavity; the right is inspection of the donor site.

On the other hand, virtual examination of patients poses a strong challenge to the clinician and limits the possibilities of a trustworthy clinical assessment. Only inspection is possible, and it is further limited by factors including inadequate compliance, low resolution cameras and unstable internet connections. In particular, oral cavity examination might be biased by inadequate compliance of patients, in particular if the lesion is localized deep in the mouth and is not easily visible using a mobile camera or a webcam; conversely, oral cavity lesions located in the anterior vestibule of the mouth, lips or the anterior tongue might be more easy to evaluate. Superficial wound inspection can be successfully performed using telemedicine, for instance, to monitor healing conditions of the skin after removal of a skin cancer or after a tissue harvest, such as a skin graft or a free vascularized flap. Using telemedicine, the patient can also be instructed by the physician on how to change complex medications. Figure 4 shows an example of wound inspection in a case of tongue reconstruction using a free forearm flap with control of the donor site.

In particular, in the postoperative follow-up of complex oncologic patients, a prominent role is played by the rehabilitation team, which is coordinated by a rehabilitation physician and includes physiotherapists. Especially during the COVID-19 lockdown, oncologic patients were at high risk of developing complications if a close follow-up was not available. Telemedicine allowed us to bring the rehabilitation team to the patient’s home, giving the patient the sensation of a closer follow-up as well as providing essential psychological support in their recovery, considering the many psycho-social aspects involved. Additionally, physiotherapists could remotely check the motion recovery, which is particularly important after a cervical lymphadenectomy has been performed, considering the neck mobility and the possibility to injure the accessory nerve, as shown in Figure 2. During the time of teleconsultation, the whole medical team consolidated around the patient, providing cohesive support and conveying an increased perception of safety in a time in which loneliness and the diminution of contacts with doctors made patients feel uncertain of their care.

We foresee that the COVID-19 pandemic will profoundly influence surgical education for young specialists and residents. In particular, university institutions have not only to focus on ensuring the quality of care and the safeguarding of doctors, but also on the need for continuous education. As health care institutions today still recommend avoiding overcrowded places, classes of live surgery events have been suspended. These factors will undoubtedly decrease possibilities of training for residents [20]. In addition, in this setting, telemedicine can provide valuable help allowing for the transmission of live surgeries by streaming, as modern devices ensure unprecedented quality of image and fluidity of video. A telemedicine interface setup for videosurgery and education was presented by the same authors in a previous experience [6]. In addition, web meetings conducted on the same platforms allow us to participate in scientific sessions where articles are reviewed and scientific threads for research are assigned to each clinician.

Several methods for teleconsultation have been presented in the literature. For instance, as described by Ghosh and colleagues [12], video sessions can be delivered using commonly available communication packages, including Skype, Zoom, Microsoft Teams, Facetime (for Apple devices) and WhatsApp. In particular, our choice of Zoom software is based on its widespread presence in the population, its simplicity of use and the excellent quality of the video stream, which translates into reduced interruptions. Additionally, Zoom permits us to identify each consultation room with an univocal code and a useful waiting room function is present, allowing us to accept the patient once the previous consultation has finished and the clinician is available, thus avoiding overlaps.

When evaluating patients’ satisfaction towards the use of teleconsultations using the already presented questionnaire, our analysis found out that patients received faster care with comparable satisfaction. We believe that patients’ perception of our telemedicine model should encourage health care institutions to pursue the development and implementation of novel telehealth platforms to improve the accessibility of care, especially in the context of a lockdown.

Even though this interface is currently very essential, since it is built on a commercially available software and uses very basic hardware, it introduces a conceptual shift which may pave the way to further improvements in terms of technology and regulations. Once mature, this system could also be routinely used for private practice outside the hospital, and software integration with applications of digital dentistry may allow us to remotely share plans for activities such as full-arch rehabilitations and orthodontics. Moreover, it may be used as a valid educational tool to teach oral surgery to younger clinicians [21,22,23,24].

Pandemics, wars and disasters represent unique challenges to health care delivery [25] and the COVID-19 emergency undoubtedly represented a catalyst for the spreading of telemedicine technology, but time is not a luxury we currently have. One of the major drawbacks is that telemedicine is quite a new field in health care, therefore strict legislation is currently lacking. The COVID-19 emergency has provided a strong impulse for an immediate implementation of such technologies, thereby widening the gap between the rapid adoption of such services into health care and their regulation. In this emergency situation, the risks related to formal inaccuracies due to the novelty of protocols, including medico-legal issues and malpractice claims, were counterbalanced by the complete lack of clinical assistance for many categories of patients. With the development of this branch of assistance, these relevant aspects will also be covered by future applications. In our protocol, which is conducted according to the Italian guidelines on the use of telemedicine [26], each patient is given a copy of the privacy policy and signed informed consent is obtained before starting teleconsultations. Moreover, when patients were discharged, they signed a module clearly stating that teleconsultations do not replace physical examinations, therefore, the patient was made aware of this concept.

Organizing clinical activity on a telemedicine platform is a very complex process, it takes time and funding sources and does not happen overnight. Clinical engineers and hospital directors should be willing to test new protocols and approve them, and new generations of doctors should become more confident with telesemeiology. In the coming years, teleconsultations will be strengthened by many technological innovations, potentially including the improvement of micro-cameras with high resolution, which could be lent to discharged patients to monitor the situation within the oral cavity using LED lightning and fiber optics. The recently introduced 5G technology may improve connectivity, decreasing latency time even in remote areas and allowing for a highly reliable communication. The further development of the Internet of Things (IoT) may enhance the possibility to connect devices normally available to patients, including blood pressure monitors and cameras, enabling clinical measures to be taken in everyday life. Apps for smartphones and tablets may be developed for a better integration of telemedicine interfaces over devices. Teleconsultations may be performed over clinically approved interfaces, allowing for data safety and achieving the same value of a physical consultation.

The COVID-19 pandemic has compelled many institutions to individually create their own protocols, both in terms of safety and remote teleassistance; however, we strongly recommend that such services are standardized and regulated, given the many clinical, technical, organizational and policy questions raised by this promising service model. The threat of a new wave of infections in the upcoming autumn should induce health care institutions to invest resources into telemedicine and organize flexible workflows without discontinuing clinical assistance to patients in the event that a new lockdown is announced.

## 5. Conclusions

The COVID-19 pandemic represents a critical time for many health care institutions. Telemedicine, if well-implemented in robust organizational models, can provide a scalable solution to overcome problems of overcrowded outpatient areas, discontinuity of care and exposure of operators to infectious risk. Our hope and belief are that such developments will continue, develop and also acquire a regulatory framework once the pandemic has ended.

## Figures and Tables

**Table 1:** “Telesemeiology checklist” with items for teleconsultations.

Questionnaire for Discharged Patients After Surgery
Facial trauma: 1. Do you have impaired vision, such as diplopia? (specific for orbital surgery)? 2. Perception of occlusion 3. Facial scars, facial nerve 4. Function (TMJ and occlusion) 5. Pain
Research for potential infection (generic information valid for any type of pathology) 1. Do you have pain? How much on a scale from 0 to 10? Where it is located? 2. Do you have fever? If yes, how much? 3. Do you have swelling? 4. Do you feel a bad taste in the mouth or bad smell in the nose?
Orthognathics: 1. Do you have reduced sensitivity of the lower lip and chin and inferior dental arch? (specific for mandibular orthognathic surgery) 2. Do you have reduced sensitivity around the infraorbital nerve area? (specific for high Le Fort I osteotomies) 3. How do you feel the occlusion? The same as after discharge or different? Do you perceive precontacts? 4. What is the extent of mouth opening? 5. Are you satisfied with the aesthetic result? 6. Check of elastodontic maneuvers 7. Postoperative photographs using standardized head position 8. Sharing of postoperative CT scan with the maxillofacial surgeons to check new bone segment position, and overall relationship condyle/fossa
Research for TMJ problems 1. What is the amount of mouth opening? 2. Do you perceive articular blocks when opening the mouth? Do you perceive clicks? 3. Do you feel pain around the ear, in correspondence to the temporomandibular joint? How much on a scale from 0 to 10? 4. Do you feel pain during chewing movements? 5. Do you perceive any swelling around the joint? 6. Sharing of postoperative CT scan if the patient received a prosthetic TMJ replacement procedure
Oncology (skin cancer and oral cancer, parotid surgery) 1. Check of the area of removal of basal cell carcinoma and referred symptoms. Healing of scar 2. Emotional status and performance status (weight loss, fatigue) 3. Mouth opening. Tongue movement. Speech function 4. Swallowing, edema, guided self-palpation of neck, scar of the neck and healing, neck mobility, impairment of accessory nerve, facial nerve and sensitive function 5. Check of donor site where a free flap was harvested (Figure 4)

**Table 2:** Satisfaction questionnaire to assess patients’ perception of teleconsultations.

Questionnaire of Satisfaction About the Use of Teleconsultation in Maintaining the Continuity of Assistance for Patients Operated in Maxillofacial Surgery Department (More than One Answer is Possible)
Do you know about the possibility to perform remote consultations using telemedicine? 1. Yes, I already tried it (2) 2. Yes, I heard about this possibility on the TV/I read something about it (1) 3. Yes, I have been told by family/friends/general practitioner (1) 4. No, I have never heard of it (0)
Do you think it is feasible with the technological devices currently owned by the average patient? 1. Yes, a smartphone with an internet connection is enough (2) 2. Only using dedicated technology and having technological skills (1) 3. No, it is limited to specialized centers (0)
Do you think that teleconsultation is a comfortable method for a consultation with the specialist that has operated on you and has followed you during hospitalization? (three options are possible) 1. No, I always prefer to come into the hospital for the traditional examination (0) 2. Yes, it would make the journey to the hospital unnecessary (1) 3. Yes, it would avoid wasting time in the waiting room (1) 4. Yes, if it avoids the need of long bureaucracy for scheduling and registering the examination (1)
Teleconsulation can replace the examination with the traditional approach for non-severe and non-urgent cases; do you agree with this method? 1. Yes (2) 2. Yes, but I prefer the traditional examination (1) 3. No (0)
Do you think privacy issues may arise? 1. No (2) 2. Yes, but I don’t care, I want to be followed by a trustworthy professional (1) 3. Yes, and I am worried for that (0)
Are you able to manage autonomously or with the help of relatives or friends the technology for teleconsultation? 1. Yes, autonomously (2) 2. Yes, under someone else’s guidance (1) 3. No, I prefer the telephone call with video (0)
Would you recommend this consultation modality? 1. Yes, a lot (2) 2. Yes, but I prefer the traditional examination (1) 3. Yes, it could replace the traditional examination in non-severe/non urgent cases (1) 4. No (0)
Do you feel safer using teleconsultations, in terms of closer follow-up by the specialist? Do you perceive the specialist somehow closer to you? 1. Yes, I have the sensation of being followed more closely (2) 2. Only as long as the teleconsultation takes place (1) 3. No (0)
